# Can a Commercially Available Smartwatch Device Accurately Measure Nighttime Sleep Outcomes in Individuals with Knee Osteoarthritis and Comorbid Insomnia? A Comparison with Home-Based Polysomnography

**DOI:** 10.3390/s25154813

**Published:** 2025-08-05

**Authors:** Céline Labie, Nils Runge, Zosia Goossens, Olivier Mairesse, Jo Nijs, Anneleen Malfliet, Dieter Van Assche, Kurt de Vlam, Luca Menghini, Sabine Verschueren, Liesbet De Baets

**Affiliations:** 1 Musculoskeletal Rehabilitation Research Group, Department of Rehabilitation Sciences, Faculty of Movement and Rehabilitation Sciences, KU Leuven, 3001 Leuven, Belgium; celine.labie@kuleuven.be (C.L.); nils.arno.andreas.runge@vub.be (N.R.); dieter.vanassche@kuleuven.be (D.V.A.); sabine.verschueren@kuleuven.be (S.V.); 2 Pain in Motion Research Group (PAIN), Department of Physiotherapy, Human Physiology and Anatomy, Faculty of Physical Education and Physiotherapy, Vrije Universiteit Brussel, 1050 Brussels, Belgium; zosia.goossens@vub.be (Z.G.); jo.nijs@vub.be (J.N.); anneleen.malfliet@vub.be (A.M.); 3 Brain, Body and Cognition, Faculty of Psychology and Educational Sciences, Vrije Universiteit Brussel, 1090 Brussels, Belgium; olivier.mairesse@vub.be; 4 PijnPraxis.be Private Practice for Pain Physiotherapy, 3970 Leopoldsburg, Belgium; 5 Unit of Physiotherapy, Department of Health and Rehabilitation, Institute of Neuroscience and Physiology, Sahlgrenska Academy, University of Gothenburg, SE-405 30 Gothenburg, Sweden; 6 Chronic Pain Rehabilitation, Department of Physical Medicine and Physiotherapy, University Hospital Brussels, 1050 Brussels, Belgium; 7 Research Foundation Flanders, 1000 Brussels, Belgium; 8 Division of Rheumatology, University Hospitals Leuven, 3000 Leuven, Belgium; kurt.devlam@uzleuven.be; 9 Skeletal Biology & Engineering Research Center, Department of Development and Regeneration, KU Leuven, 3000 Leuven, Belgium; 10 Department of General Psychology, University of Padova, 35131 Padova, Italy; luca.menghini-1@unitn.it; 11 Human Inspired Technology Research Centre, University of Padova, 35131 Padova, Italy; 12 Department of Physical and Rehabilitation Medicine, University Hospitals Leuven, 3212 Leuven, Belgium; 13 Leuven Algologic Center, University Hospitals Leuven, 3212 Leuven, Belgium

**Keywords:** knee osteoarthritis, sleep, insomnia, wearable, polysomnography, accuracy

## Abstract

**Highlights:**

**What are the main findings?**

- Fitbit Sense demonstrates high accuracy and sensitivity for detecting sleep in individuals with knee osteoarthritis and insomnia.
- The device shows limited ability to differentiate quiet wakefulness from sleep and less precision in classifying specific sleep stages.

**What is the implication of the main finding?**

- Fitbit Sense can be a useful complementary tool for monitoring general sleep duration, timing, and regularity in this population.
- Sleep stage and fragmentation data should be interpreted with caution, as agreement with polysomnography decreases under more disrupted sleep conditions.

**Abstract:**

Sleep is a vital physiological process for recovery and health. In people with knee osteoarthritis (OA), disrupted sleep is common and linked to worse clinical outcomes. Commercial sleep trackers provide an accessible option to monitor sleep in this population, but their accuracy for detecting sleep, wake, and sleep stages remains uncertain. This study compared nighttime sleep data from polysomnography (PSG) and Fitbit Sense in individuals with knee OA and insomnia. Data were collected from 53 participants (60.4% women, mean age 51 ± 8.2 years) over 62 nights using simultaneous PSG and Fitbit recording. Fitbit Sense showed high accuracy (85.76%) and sensitivity (95.95%) for detecting sleep but lower specificity (50.96%), indicating difficulty separating quiet wakefulness from sleep. Agreement with PSG was higher on nights with longer total sleep time, higher sleep efficiency, shorter sleep onset, and fewer awakenings, suggesting better performance when sleep is less fragmented. The device showed limited precision in classifying sleep stages, often misclassifying deep and REM sleep as light sleep. Despite these issues, Fitbit Sense may serve as a useful complementary tool for monitoring sleep duration, timing, and regularity in this population. However, sleep stage and fragmentation data should be interpreted cautiously in both clinical and research settings.

## 1. Introduction

Sleep is a universal behavior essential for recovery and health regulation. In chronic pain, it not only reflects symptom burden but also actively contributes to pain persistence and impaired functioning [1]. Knee osteoarthritis (OA) is a leading cause of chronic pain and disability [2], with increasing evidence linking sleep disturbances to poorer disease outcomes [3,4,5,6,7,8]. Monitoring sleep is necessary to better understand these associations, and although research on self-reported sleep in OA populations is well established, studies using objective sleep measures remain limited [9,10,11]. Preliminary evidence suggests that prolonged nocturnal awakenings are predictive of next-day pain intensity [12], and alterations in sleep staging have also been documented [13,14,15]. Moreover, individuals with knee OA and comorbid insomnia display irregular sleep–wake patterns, further disrupting endogenous activity–rest rhythms [16].

This limited research on objective sleep outcomes in knee OA highlights the need for accessible, reliable sleep tracking tools for research and clinical practice [17,18,19,20]. While PSG is the gold standard, it requires a costly, burdensome setup in-laboratory or a home-based setup and trained sleep scorers. Research-grade actigraphy devices are recommended for estimating sleep in individuals with insomnia [21]. They detect sleep–wake states but have limited ability to assess sleep stages [22]. Consumer sleep tracking (CST) devices, including smartwatches, finger rings, and smart straps, offer a scalable and non-invasive alternative for monitoring sleep in real-world settings. These devices are popular because of their lower cost and greater acceptability. Using sensors, such as accelerometry and photoplethysmography-based sensors, CST devices estimate sleep outcomes, potentially providing an affordable, user-friendly solution for large-scale sleep data collection in free-living conditions [23]. For individuals with chronic pain, CST may support self-management by increasing awareness of behavioral sleep patterns and their interaction with symptom fluctuations. Clinicians may benefit from these data to tailor interventions and monitor treatment responses over time. Moreover, CST’s ability to concurrently capture other behavioral health-related indicators, such as physical activity and stress-related metrics, supports a more integrated understanding of lifestyle factors contributing to pain and recovery [24,25]. These devices not only enable continuous monitoring but also hold promise for advancing personalized healthcare applications, particularly in the prevention and management of chronic conditions such as knee OA and insomnia [26,27,28].

Despite these advantages and opportunities, the accuracy and performance of CST devices remain largely unknown, especially in clinical populations, emphasizing the necessity for thorough evaluation in research and practice [20]. Standardized protocols for evaluating CST device performance have recently been developed [29], and studies have assessed their accuracy across diverse populations, including healthy individuals [30,31] and those with sleep disorders [32,33], Huntington’s disease [34], and psychiatric conditions [35,36]. To date, no research has investigated the quality performance of CST devices in populations with chronic pain conditions. In individuals with knee OA, pain-related sleep disruptions, irregular sleep–wake cycles, and altered movement patterns may further compromise CST accuracy, underscoring the need for population-specific performance testing. This study addresses this gap by assessing the performance of the Fitbit Sense for nighttime sleep outcomes compared with home-based polysomnography in individuals with knee OA and comorbid insomnia.

## 2. Materials and Methods

Data were collected from a large randomized controlled trial (RCT) investigating the effects of cognitive–behavioral therapy for insomnia integrated with evidence-based physiotherapy in people with knee OA and insomnia (ClinicalTrials.gov NCT05387473—registration date 24 May 2022) [37]. For this study, data from the screening procedure and post-intervention assessment were used. This study was approved by the ethics committees of the University Hospitals Leuven and the University Hospital Brussels (S66306) and conducted in compliance with the Helsinki Declaration [38]. All participants provided written informed consent before enrolment in this study. The analyses were conducted following Menghini et al.’s standardized framework and guidelines for CST performance evaluation [29,39]. This study’s findings were reported and written up following the guidelines provided by the 22-item checklist from the STROBE Statement for cross-sectional studies [40].

### 2.1. Participants

Participants were recruited from the community areas around Brussels and Leuven (Belgium) between May 2022 and August 2024. Participants were initially screened by phone to assess their eligibility. Included participants were adults aged ≥ 45 years reporting average knee pain ≥ 3/10 on most days of the week for at least 3 months, with KOA as classified by the American College of Rheumatology [41], and diagnosed with insomnia using the Diagnostic and Statistical Manual of Mental Disorders, fifth Edition criteria [42]. Exclusion criteria included: (1) body mass index > 30 kg/m^2^; (2) knee replacement; (3) known pre-diagnosed sleep disorder such as sleep apnea or periodic limb movement disorders; (4) rheumatological or neurological conditions; (5) cancer diagnosis or treatment in the last year; (6) receiving cholinesterase inhibitors; (7) receiving supervised knee exercise therapy or knee joint infiltration in previous 6 months; (8) presence of an external factor limiting opportunity to sleep (e.g., newborn). Participants underwent at-home polysomnography to screen for intrinsic sleep disorders such as sleep apnea or periodic limb movement disorders, which would result in exclusion from the trial. However, for this study, all screened individuals at the moment of the screening procedure were considered for inclusion, even if a sleep disorder was detected during the PSG screening night. Furthermore, data collected during the post-intervention follow-up assessment were also considered for this study.

### 2.2. Measurements

#### 2.2.1. Procedure and Data Collection

The sleep assessment was performed during one night between 30 August 2022 and 21 November 2024, and was part of the at-home screening procedure or post-intervention assessment of the original RCT. An experienced researcher—specifically trained in installing the CST and PSG devices—visited the participants’ homes in the evening hours to install both devices and inform the participants of their appropriate use. Participants were sleeping with a CST device, the Fitbit Sense (Fitbit^®^, San Francisco, CA, USA—Firmware and software version: FITBIT OS 5.3.1—Version 44.128.6.17) and simultaneously underwent at-home PSG measurement using the Alice PDX portable sleep diagnostic system (Philips Respironics, Murrysville, PA, USA). Both devices were used and worn as per the manufacturer’s instructions and specifications. The device was placed on the non-dominant wrist and a finger’s width above the styloid process of the ulna, and was worn until the next morning.

For this one-night assessment, the timing and duration of their sleep were self-selected and unrestricted, and participants were instructed to adhere to their habitual sleep schedule. Participants were asked to refrain from caffeine after lunch, smoking in the evening, and alcohol consumption as well as intensive sport for the whole day before the evaluation. Additionally, participants were asked to take their regular medications and not to initiate new pharmacological treatments on the day of the recording.

Around the time of the sleep assessment, participants received an invitation via email to digitally complete self-reported questionnaires for sociodemographic, pain-related, and sleep-related factors using the UZ Leuven REDCap online system (REDCap 12.4.13; Vanderbilt University, Nashville, TN, USA) [37].

#### 2.2.2. Data Processing

The time in bed (TIB) period was defined as the interval between lights-off (participant getting in bed with the intention to sleep—start initiated by the participant pressing a button on the PSG) and lights-on times (participant getting out of bed with the intention to start the day—end initiated by turning the PSG device off). These events were primarily determined by the participant pressing a button on the PSG; however, when the button was not pressed, an alternative approach was used. Specifically, lights-off was estimated based on the PSG-detected transition from standing to lying down closest to the sleep diary-reported time (used on 34 nights, compared with 30 nights where the button was pressed). Similarly, lights-on was determined by the transition from lying down to standing when the button press was missing (used on 8 nights, compared with 56 nights where the button was pressed). The exported epochs and the TIB period were manually synchronized to the PSG’s lights-off/on times.

Fitbit Sense Sleep Outcomes and Processing

The Fitbit Sense was selected for this clinical validation study based on its superior performance compared with other Fitbit models [30,43], the unavailability of the Fitbit Sense 2 at the time, and cost considerations that allowed for an adequate sample size within the original RCT design. Fitbit Sense tracks motion, heart rate, heart rate variability, and respiratory rate via a 3-axis accelerometer (sampling rate of 100 Hz) and optical plethysmography, respectively (sampling rate disclosed by Fitbit). Collected sensor data are processed via a device- and company-specific algorithm [44]. The Fitbit Sense’s proprietary sleep stage classification algorithm integrates motion, heart rate variability, and respiratory rate data to provide information on sleep versus wake states and sleep staging (i.e., wake; light sleep as equivalent of PSG N1 + N2; deep sleep as equivalent of PSG N3; and REM sleep). These data can be extracted from the Fitbit web interface (Small Steps Labs LLC, San Diego, CA, USA) once the device is synchronized with the interface. For this study, the algorithm sensitivity option for the ‘normal setting’ was selected. No additional custom thresholds or smoothing beyond the default Fitbit Sense algorithm settings were applied. For each recording, Fitabase provides sleep stage data, including timestamps, in 30 s epochs to allow epoch-by-epoch (EBE) analysis.

PSG Sleep Outcomes, Processing, and Scoring

PSG was used as a gold standard reference measure to evaluate sleep outcomes obtained by the CST device. The overnight home-based PSG assessment was conducted in a free-living monitoring environment, where factors such as sound and temperature were not controlled. The following parameters were recorded: electroencephalography (EEG) (electrodes placement on frontal midline (Fz) and central midline (Cz), each referenced to the left mastoid (M1)), electrooculography (EOG) (left and right), electromyography (EMG) chin and tibialis (left and right), oronasal flowmetry (pressure-based airflow with snore detection through a nasal cannula and thermistor), oxygen saturation (SpO2, finger probe, Oximetry board Nonin), pulse rate (from the oximeter probe) and body position (up, supine, prone, left, and right). The PSG electrode sites were measured and applied according to the International 10–20 System of EEG Electrode Placement. PSG data were collected and recorded at a sampling rate of 250 Hz, with bandpass filters applied as follows: ECG (0.318 Hz to 81 Hz), EEG (0.318 Hz to 35 Hz), and EMG (9.7 Hz to 86 Hz). After visual inspection of recordings, manual scoring software (Sleepware 3G, Philips Respironics, Murrysville, PA, USA) was used for scoring sleep versus wake states, sleep stages, and events following the American Academy of Sleep Medicine Manual [45] (version 2.4 released April 2017). Each 30 s epoch of PSG data was categorized as wake, N1, N2, N3, or REM sleep by two independent raters, who were blinded to the CST device’s output [39]. The inter-rater reliability, measured by Cohen’s Kappa, was 68%. Discrepancies were addressed through discussion, with a third rater consulted for any unresolved disagreements. Raw data of scored recordings were exported as xls-files to allow EBE analysis. For the purpose of this study, outcomes of interest were the same sleep outcomes as derived from the Fitbit Sense (TST, SE, SOL, and WASO), and duration (in minutes) spent for each sleep stage. PSG stages N1 and N2 were merged into one category representing light sleep in the device, and N3 was referred to as deep sleep.

### 2.3. Data Selection

Data from participants who met the inclusion criteria from this study and with no missing sleep data were used. PSG recordings were defined as failed if scoring was not possible because of poor signal quality, loosening of material, other technical issues, or when no signals were recorded at all. For the device, nights with poor signal quality (no sleep stages output) or nights with no recorded sleep episode data were excluded. Available eligible nights from screening and post-intervention assessment were selected, allowing multiple nights per participant.

### 2.4. Statistical Analysis

Outcomes were calculated based on the recommended standards for evaluating CST devices, utilizing the open-source R code from Menghini et al. (2021) [29]. The performance evaluation followed this standardized framework, which included discrepancy analysis, Bland–Altman plots, and EBE analysis. EBE analysis was conducted using 30 s epochs, following the temporal synchronization of Fitbit Sense with PSG data, with all analyses limited to the lights-off-to-lights-on period. The sample size was based on the number of nights for which valid, concurrent data were available from both Fitbit Sense and PSG. A significance threshold of 0.05 was applied for all tests. All analyses were carried out using R software version 4.3.2 (R Foundation for Statistical Computing). No log transformations or normalization procedures were applied to data before analysis.

#### 2.4.1. Descriptives

Descriptive statistics were generated for all demographic and baseline characteristics. The normality of their distributions was assessed using histograms, Q–Q plots, and Kolmogorov–Smirnov tests. Percentages were calculated for categorical variables. For the self-reported questionnaires and sleep outcomes, the mean (standard deviation) was reported for normally distributed data, while the median (interquartile range) was reported for non-normally distributed data. A threshold of 1.5 times the interquartile range was applied to the discrepancies between Fitbit Sense and PSG across all sleep outcomes to identify outliers.

#### 2.4.2. Discrepancy Analysis and Bland–Altman Plot

Discrepancy analysis evaluated bias and limits of agreement (LOAs) between sleep outcomes derived from Fitbit Sense and PSG. Bias was determined by subtracting PSG values from Fitbit Sense values, where positive differences indicate overestimation by Fitbit Sense and negative differences indicate underestimation. These discrepancies, assessed at the individual level, estimate systematic bias and random measurement error (95% LOAs) using Bland–Altman plots. The analysis involved testing key assumptions: (1) whether bias remains constant or varies with measurement size (proportional bias), (2) whether the variability of differences is uniform (homoscedasticity) or changes in measurement size (heteroscedasticity), and (3) whether differences follow a normal or non-normal distribution. To assess proportional bias and heteroscedasticity for each sleep outcome, linear regression was used. When proportional bias was significant (*p* < 0.05), indicating changes in bias with measurement size, discrepancies were adjusted accordingly by modeling the bias as a linear function of PSG-derived values, allowing for the estimation of conditional bias at specific PSG levels rather than assuming a constant mean difference across the measurement range. Similarly, if heteroscedasticity was detected, LOAs were modeled based on measurement size to account for variability in the differences. The significance of Fitbit Sense over- and underestimation was assessed based on the 95% confidence intervals of the bias.

#### 2.4.3. EBE Analysis

To assess performance metrics for each sleep stage, the outcomes were dichotomized, comparing the sleep stage of interest against all others. Error matrices comparing the Fitbit Sense outcomes to PSG were generated for each participant and the whole group. Specifically, the number of epochs classified as light sleep, deep sleep, REM sleep, or wake by both methods was computed. The average proportion of correct (sensitivity) and incorrect (specificity) Fitbit Sense classifications relative to PSG classifications was reported using a proportional error matrix, along with the corresponding standard deviation and 95% confidence intervals. The traditional definitions of sensitivity (i.e., the ability to correctly classify sleep epochs) and specificity (i.e., the ability to correctly classify wake epochs) have been adapted to account for multi-stage sleep classification [20,39]. Instead of a binary sleep–wake categorization (0 = wake, 1 = sleep), classifications were expanded to include four distinct stages: 0 = wake, 1 = “light” sleep, 2 = “deep” sleep, and 3 = REM sleep. Sensitivity was calculated for each sleep stage, representing Fitbit Sense’s ability to correctly identify a given PSG stage (wake, “light,” “deep,” or REM), while specificity measured its ability to correctly classify all other PSG stages. For further details on these definitions, see Menghini et al. [29]. Cohen’s kappa (κ), which measures classification agreement beyond chance on a scale from 0 to 1, and the prevalence-adjusted bias-adjusted kappa (PABAK) coefficient were computed for each sleep stage. Additionally, the positive predictive value (the proportion of epochs identified as a target stage by Fitbit Sense that are also classified as that stage by PSG) and negative predictive value (the proportion of epochs not classified as a target stage by Fitbit Sense that are also not classified as that stage by PSG) were calculated for each sleep stage.

#### 2.4.4. Sensitivity Analysis

Several sensitivity analyses were conducted to assess the robustness of the findings. The first evaluated the performance and accuracy of this study device using all available data points, including outliers. The second focused on data from participants with only a single night of recordings. The third compared baseline demographic characteristics between included and excluded participants, using either a t-test or its nonparametric equivalent, depending on data distribution.

## 3. Results

### 3.1. Characteristics of Participants

**Figure 1:**
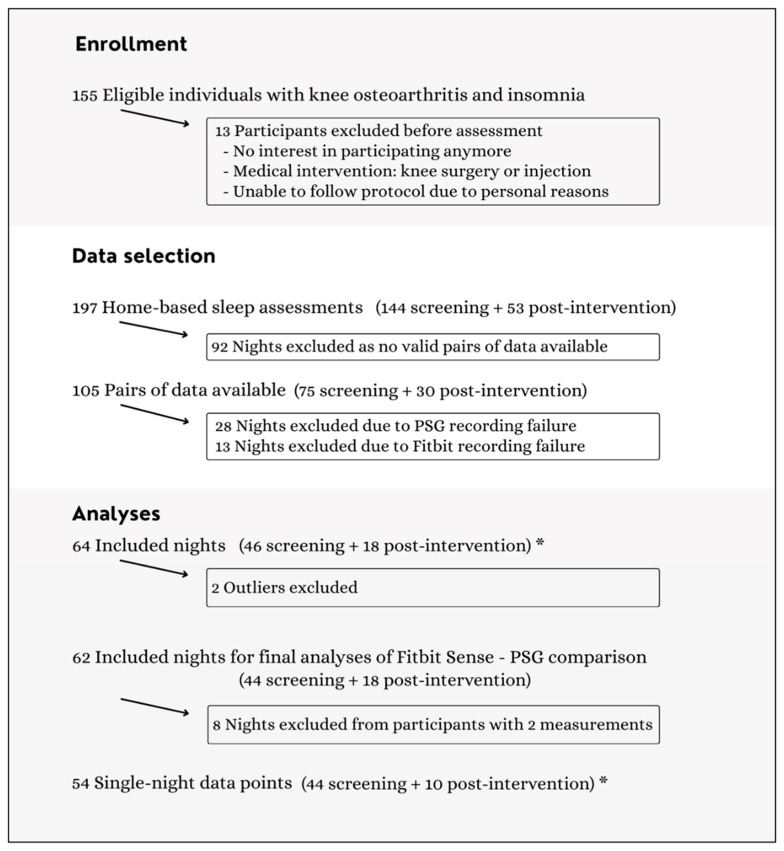
Flow chart illustrating the study enrollment, data selection, and analyses. * Additional analyses are available in Supplemental Material.

A total of 155 eligible individuals with knee OA and insomnia were identified for this study. Of these, 13 participants withdrew before assessment due to lack of interest, medical interventions, or inability to follow the study protocol (e.g., time constraints). After checking for valid concurrent nights, 105 home-based sleep assessments remained. During data processing, 41 nights were excluded because of recording failures from the PSG or study device. From the remaining 64 nights, a total of 14 outliers were identified using the predefined criterion of 1.5 times the interquartile range for the discrepancies between the device and PSG across all sleep outcomes. Outliers were further examined through visual inspection of data, allowing for a more comprehensive assessment. Two participants accounted for half of these outliers and were removed from the analysis because of multiple unlikely values. All other data points were cross-checked with original records, considered valid, and included in the final analysis. The final number of nights included for the Fitbit–PSG comparison was 62 nights (44 from the baseline screening and 18 from the post-intervention assessment) from 53 participants (Figure 1).

Participants were predominantly older adults (61 years ± 8.2; 98.1% White-Caucasian; 60.4% female; BMI of 24.7 ± 2.6 kg/m^2^) with long-standing knee pain (mean duration: 9.7 ± 9.7 years) of moderate severity (NRS: 5.2 ± 1.9). A total of 86.5% experienced nighttime pain, and 84.9% of participants reported one or more comorbidities other than knee pain and insomnia. Average insomnia duration was 11.1 ± 11 years, with 96.1% reporting poor sleep quality (PSQI > 5) and 66.7% meeting criteria for clinical insomnia (ISI ≥ 15). The detailed demographics and baseline characteristics of the 53 participants studied are presented in Table 1. PSG-derived sleep outcomes are reported in Table 2. The number of participants for each outcome measure is provided in Supplementary Material Table S1.

### 3.2. Fitbit Sense–PSG Comparison

#### 3.2.1. Discrepancy Analysis

**Figure 2:**
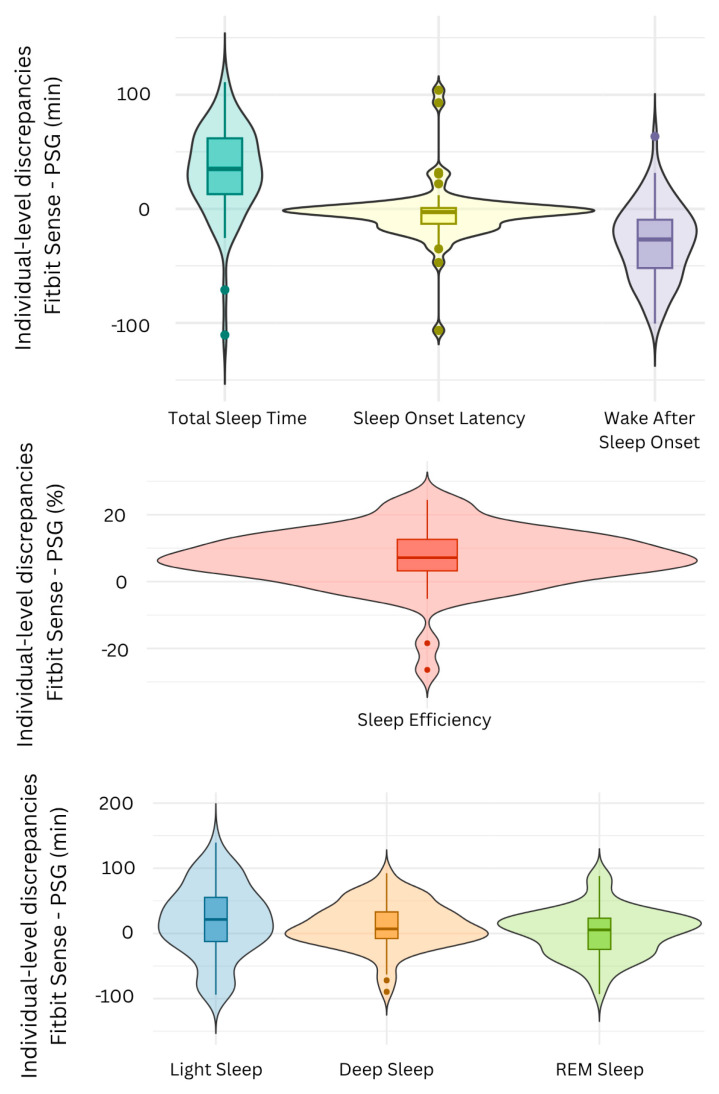
Distribution of individual-level discrepancies between Fitbit Sense and PSG for each sleep outcome. The boxplot elements include a colored horizontal line indicating the median, a box representing the interquartile range (first to third quartile), and whiskers extending to the upper and lower adjacent values. These elements are consistently applied across all violin plots, which further illustrate the data distribution by showing the density of values across different ranges. Data points beyond 1.5 times the interquartile range are represented by colored dots, highlighting potential outliers within the sample. PSG = Polysomnography; REM = Rapid-eye-movement.

Individual-level discrepancies were calculated by subtracting PSG-derived outcomes from Fitbit-derived outcomes for each subject. Figure 2 presents the summary statistics and distribution for each sleep outcome.

Group-level discrepancies were assessed using mean bias (Fitbit Sense–PSG), mean absolute difference, and standard error. A summary of group-level differences between the Fitbit Sense and PSG is provided in Table 3.

**Figure 3:**
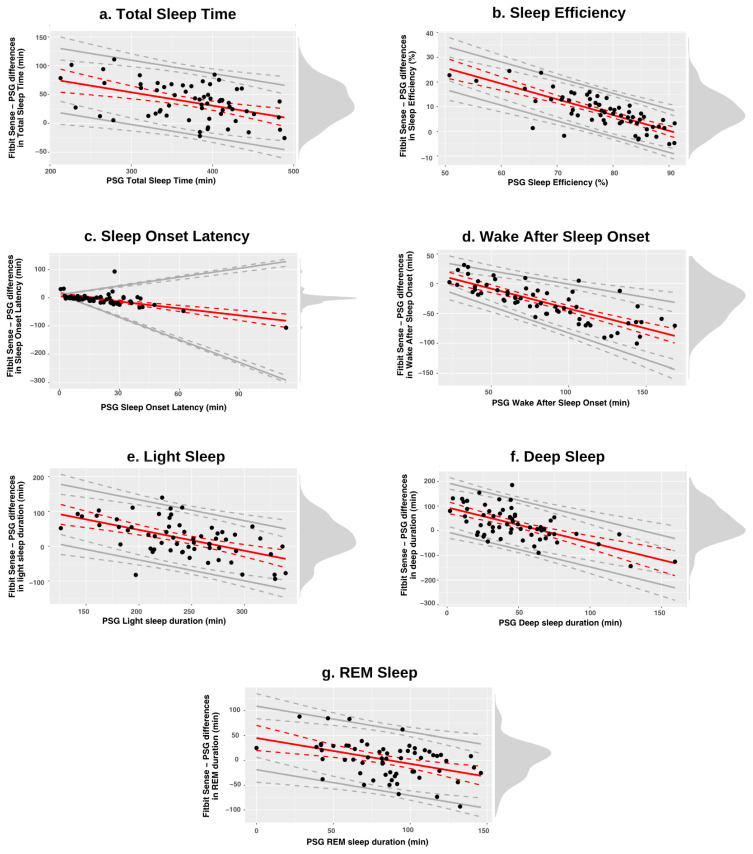
Bland–Altman plots of the sleep outcomes for the Fitbit Sense–PSG comparison for each sleep stage. Red solid lines indicate bias, whereas gray solid lines indicate the 95% LOAs, both with their 95% CIs (dotted lines). Black points indicate individual observations, and the density diagram on the right side of each plot represents the distribution of the Fitbit Sense–PSG differences. The zero point on the *y*-axis denotes perfect agreement between the two methods, while values above and below zero indicate overestimation and underestimation, respectively, in comparison to PSG. A diagonal trend in the mean bias line indicates a considerable proportional bias, whereas diverging limits of agreement suggest notable heteroscedasticity. PSG = Polysomnography; REM = Rapid-eye-movement.

The results of Bland–Altman analysis showed a significant proportional bias across all sleep outcomes, with the magnitude and direction of this bias varying according to the range of PSG-derived values (Table 4). These biases were modeled using linear regression, and the corresponding Bland–Altman plots illustrate how the bias changes across the range of measurements (Figure 3).

*TST and SE* exhibited significant negative proportional biases, indicating that the device tended to overestimate these outcomes, particularly at higher PSG-derived values. For TST, the overestimation decreased as PSG values surpassed 450 min, beyond which the bias became statistically non-significant (Figure 3a). At an average PSG-derived TST of 373 min, the device overestimated TST by 37.58 min. Similarly, SE was overestimated for nights with high PSG-derived SE values, with the bias becoming non-significant when PSG-based SE exceeded 85% (Figure 3b). A negative proportional bias was also found for *SOL and WASO* (Figure 3c,d). However, whereas TST and SE discrepancies showed larger deviations from zero for smaller PSG-derived values, the opposite pattern was found for SOL and WASO, showing larger deviations for longer PSG-derived SOL and WASO values. The bias became statistically non-significant for lower PSG-derived values (approximately 13 min and 50 min or less, respectively). Notably, the variability in differences decreased as PSG-derived SOL and WASO values declined (i.e., heteroscedasticity), suggesting improved agreement at lower SOL and WASO levels. *Light sleep* duration was also overestimated by the device, particularly when PSG-derived values were below 250 min. For values exceeding this threshold, the bias became non-significant (Figure 3e). However, the wide LOAs associated with this measure indicate considerable variability and poor agreement with PSG, highlighting the need for caution when interpreting light sleep estimates. For both *deep sleep and REM sleep* durations, the average bias appeared to be close to zero near the mean PSG-derived values. However, the bias became more evident at the lower and upper extremes of the PSG measurement range (Figure 3f,g).

#### 3.2.2. Epoch-by-Epoch Analysis

Light sleep (73 ± 10%) and REM sleep (60 ± 27%) showed the highest correct classification rates. A substantial proportion (37 ± 15%) of wake epochs were misclassified as light sleep, indicating difficulties for Fitbit Sense in distinguishing wakefulness from light sleep. Furthermore, 46 ± 25% of deep sleep epochs and 33 ± 25% of REM sleep epochs were misclassified as light sleep, indicating difficulties in differentiating between those stages too.

The detailed group-level proportional error matrix from the EBE analysis is presented in Table 5 and provides insights into systematic misclassifications and the overall performance of the classification. Additionally, the absolute error matrix, which considers the total epoch count in a given classification category without differentiating between subjects, is provided in the Supplementary Material Table S2.

The Fitbit Sense demonstrated distinct performance patterns across sleep stages when evaluated against PSG using stage-specific group-level EBE metrics.

Wake Detection

Fitbit Sense demonstrated high accuracy (85.76 ± 5.49%) and very high specificity (95.95 ± 2.9%) for wake detection, indicating reliable identification of non-wake epochs. However, sensitivity was moderate (50.96 ± 15.46%), reflecting a significant under-detection of wake, often misclassified as light sleep. The agreement with PSG was moderate to substantial (κ = 0.51 ± 0.13; PABAK = 0.72 ± 0.11), confirming reliable wake detection despite this underestimation.

Light Sleep

Light sleep showed the lowest accuracy (67.22 ± 7.85%) and specificity (62.62 ± 13.32%), indicating frequent misclassification of other stages as light sleep. While sensitivity was highest for light sleep (72.50 ± 9.98%), the positive predictive value was moderate (66.51 ± 11.98%), suggesting overestimation of light sleep at the expense of deeper stages. The agreement with PSG was low (κ = 0.34 ± 0.15).

Deep Sleep

Deep sleep detection had high accuracy (87.65 ± 4.57%) and specificity (91.76 ± 4.41%), but low sensitivity (48.75 ± 27.24%), showing frequent misclassification of deep sleep as light sleep. The positive predictive value was low (39.65 ± 25.94%), highlighting substantial false positives. Despite low κ (0.34 ± 0.23), prevalence-adjusted agreement was substantial (PABAK = 0.75 ± 0.10).

REM Sleep

REM sleep accuracy was high (85.23 ± 6.02%) with good specificity (91.76 ± 4.41%). Sensitivity was moderate (60.41 ± 26.51%), indicating a notable proportion of REM epochs were misclassified, mostly as light sleep. The positive predictive value was modest (56.62 ± 18.09%) and agreement metrics showed moderate reliability (κ = 0.47 ± 0.23; PABAK = 0.70 ± 0.12).

Details of the group-level EBE metrics are provided in Table 6. These EBE metrics are reported as averages ± SD across subjects. Detailed visualizations of individual-level EBE metrics are available in the Supplementary Material Figure S1. Additionally, group-level EBE metrics based on the absolute error matrix, which considers the total epoch count per condition without differentiating between subjects, are also available in the Supplementary Material Table S3.

### 3.3. Sensitivity Analyses

The results of the first sensitivity analysis, which examined performance and accuracy over 64 nights including all data points with outliers, are presented in Supplementary Material Figure S2 and Table S4–S6. The second sensitivity analysis, based on 54 nights from participants with only a single night of data, is described in Supplementary Material Figure S3 and Table S7–S9. The third sensitivity analysis compared baseline characteristics between included and excluded participants. No significant demographic differences were found, except for age, with excluded participants being slightly younger. These results are available in Supplementary Material Table S10.

## 4. Discussion

This is the first study to assess the performance of the Fitbit Sense for detecting sleep outcomes compared with PSG under free-living conditions in individuals with knee OA and insomnia. The device demonstrates strong sleep vs. wake detection, with high accuracy (85.76%) and sensitivity (95.95%) for identifying sleep epochs. These results align with prior performance evaluation studies [43,46,47] and support its utility for general sleep monitoring in this clinical population. The high sleep sensitivity suggests the device reliably detects sleep periods, making it suitable for tracking overall sleep duration and parameters such as timing, regularity, and chronotype in individuals with knee OA and insomnia.

Our results also show Fitbit Sense–PSG agreement varied depending on the size of the measurement. The agreement was highest on nights with longer sleep (TST > 450 min), more time asleep (SE > 85%), faster sleep onset (SOL < 13 min), and fewer awakenings (WASO < 50 min). Under these conditions, discrepancies were minimal or non-significant, suggesting robust performance when sleep is less fragmented. These findings align with previous Fitbit–PSG comparisons in healthy [30,31] and clinical populations, including insomnia [32], obstructive sleep apnea [33], Huntington’s disease [34], major depressive disorders [36], and psychiatric disorders [35]. As in prior studies, sleep duration, light sleep, and REM were overestimated, while the duration of nocturnal awakening was underestimated. However, unlike earlier studies reporting underestimation of deep sleep [30,31,32,33,34,35,36], the device overestimated this stage. Similar overestimations of deep sleep by other CSTs [30,31] suggest such discrepancies may reflect device-specific algorithms or population-specific factors.

Despite strengths, the device had limited wake detection accuracy with a moderate wake sensitivity of 50.96%. While performing better than earlier Fitbit models (13.10–69.76%) [30,32,33,47,48,49,50], this highlights a persistent challenge for CSTs: distinguishing quiet wakefulness from sleep. Reliance on actigraphy and heart rate sensors likely contributes to the misclassification of motionless wake as sleep. When compared with the newer Fitbit Sense 2 in young healthy adults, two studies reported modest improvements in sleep stage sensitivity. Sensitivities for light, REM, and deep sleep using Sense 2 were 77.34% and 78.00%, 67.10% and 61.70%, and 68.12% and 67.30%, respectively, versus 72.50%, 48.75%, and 60.41% in our study [46,51]. These differences may reflect updated algorithms and enhanced sensors, including improved heart rate variability and skin temperature tracking, possibly supporting more accurate sleep staging. These differences may also reflect population-related variability, as our sample differs from the healthy young adults assessed in prior studies. Sleep staging and wearable sensor performance may vary across populations with differing health conditions and sleep disturbances, influencing the accuracy of CSTs, which generally perform better in healthy individuals. For example, Kang et al. (2017) reported lower accuracy of the Fitbit Flex in individuals with insomnia compared with good sleepers, suggesting sleep disturbances may impair device performance [52]. Similarly, a study using the same CST as ours found slightly lower agreement with PSG in individuals with psychiatric disorders, with light, deep, and REM sleep sensitivities of 68%, 44%, and 49%, respectively, less than those observed in our sample [35].

In individuals with knee OA, pain-related awakenings involving subtle repositioning due to discomfort may lack sufficient wrist movement and remain undetected by CSTs [8]. Pain- and insomnia-related arousals (brief EEG-defined awakenings) may fall below CST sensitivity thresholds, leading to underreported nocturnal awakenings and fragmented sleep, key features of knee OA-related sleep disturbance [13,14,15,53]. Similarly, individuals with insomnia often lie awake without movement, making these wakeful periods prone to misclassification as sleep. Beyond the sleep–wake distinction, CSTs face difficulties in accurately identifying sleep staging. The device exhibited high specificity across stages, but lower sensitivity for wake, deep, and REM sleep, with many epochs misclassified as light sleep. This imprecision complicates clinical interpretation, potentially masking true sleep disturbances in individuals with knee OA and insomnia. Pain in knee OA may fragment sleep cycles via micro-arousals, disrupting stage continuity. Consequently, CST algorithms may label ambiguous epochs as light sleep. Since these devices already have difficulties detecting REM and deep sleep in healthy individuals, underrepresentation of these stages in individuals with knee OA and insomnia could exacerbate the overestimation of light sleep and underestimation of restorative sleep.

### 4.1. Clinical Implications

This study underscores the clinical relevance and limitations of the Fitbit Sense for sleep monitoring in individuals with knee OA and comorbid insomnia. The device offers an accessible way to monitor sleep-derived parameters, including sleep duration, timing, regularity, and chronotype, which are increasingly recognized as modifiable factors influencing pain-related outcomes. For clinicians and researchers, such data may help contextualize fluctuations in symptom severity and functional limitations, offering a more continuous and objective view compared with self-reported questionnaires, which may be subject to recall bias and limited by periodic assessments. For example, patterns of irregular sleep timing or short sleep duration may inform the delivery of components within cognitive–behavioral therapy for insomnia (CBT-I), such as sleep scheduling or stimulus control, and may also help guide behavioral strategies in individuals with chronic pain, where improving sleep regularity and alignment could contribute to better symptom management. For patients, increased visibility into their sleep patterns may foster awareness of unhelpful behaviors (e.g., inconsistent sleep schedules or extended time in bed) and encourage sleep-promoting practices, such as adherence to regular routines or time in bed restrictions. This feedback loop between data and behavior may be relevant in populations where pain-related hyperarousal (i.e., difficulty falling asleep due to pain-induced arousal) or unhelpful beliefs about sleep (e.g., unrealistic expectations such as needing eight hours of sleep to function the next day). However, the device’s reduced accuracy in detecting wakefulness, estimating sleep efficiency, and classifying sleep stages suggests it is not suitable for diagnostic decision-making or detailed assessment of sleep architecture. Instead, CSTs should supplement clinical assessments, subjective evaluation, and validated tools such as sleep diaries or, when needed, polysomnography (PSG) [54].

These limitations emphasize the importance of user education and careful interpretation. The observed variability in device accuracy across the magnitude of nighttime sleep outcomes (e.g., duration of nocturnal awakenings) suggests that the device may be less precise for individuals with more severe sleep disruptions. Clinicians and users should consider this potential limitation, as accuracy may decrease with more extreme sleep patterns, affecting the interpretation of results. Without guidance, CST data may result in false reassurance or increased anxiety, potentially exacerbating both sleep disturbances and pain. Incorporating patient-reported outcomes alongside objective data enables clinicians to identify and interpret discrepancies such as reports of non-restorative sleep despite apparently adequate total sleep time, within the patient’s broader clinical picture, as CSTs are unable to capture the complex and context-dependent factors that influence sleep quality and quantity. Understanding the limitations of CSTs is essential for effective and appropriate use. Importantly, the continuous feedback from sleep trackers can trigger orthosomnia (increased sleep-related anxiety that may lead to insomnia), especially in patients already prone to hyperarousal [55]. Heightened focus on nightly data may reinforce excessive monitoring and cognitive preoccupation with sleep. To prevent this, clinicians should encourage adaptive interpretation of CST data, emphasizing longer-term trends rather than nightly fluctuations. This should occur within a biopsychosocial framework that considers emotional regulation, physical activity, and cognitive patterns contributing to sleep and pain outcomes.

### 4.2. Future Research

Future research should include normative comparisons for disorder-specific effects and assess intra-individual variability over multiple nights to detect natural fluctuations and treatment-related changes. Research involving larger, more diverse cohorts with rigorous control of potential confounders—including comorbidities (e.g., obesity and chronic diseases), medications affecting sleep architecture and behavior, and environmental factors such as geographical living environments and household context—is essential to better understand their impact on wearable device performance and sleep outcomes.

Longitudinal studies are needed to evaluate CST stability during interventions, especially in older adults with knee OA. CST–PSG agreement should be assessed based on clinically meaningful thresholds rather than perfect agreement, focusing on trend detection. Physiological states, including inflammation, autonomic dysregulation, or metabolic shifts, may influence CST accuracy by altering sleep architecture or sensor responsiveness. Given the high rate of multimorbidity in knee OA populations [56,57], comorbidities such as cardiovascular disease, diabetes, and depression should be accounted for in device validation.

Despite limitations, CSTs support patient engagement and self-monitoring, potentially improving adherence to behavioral sleep interventions. Future studies should examine how patients interpret CST data, especially in cases of mismatch with subjective experience, to minimize misperceptions, reduce orthosomnia risk, and optimize clinical integration [58].

### 4.3. Strengths and Limitations

This study presents several strengths. It focuses on a clinically relevant and understudied population and evaluates sleep tracking under free-living conditions using gold-standard home PSG. The methodology adhered to established scoring guidelines, including double scoring of PSG data, and examined both global and stage-specific sleep parameters.

However, several limitations should be acknowledged. The findings are specific to the Fitbit Sense and its proprietary algorithms, which lack transparency and limit both generalizability to other consumer sleep technology (CST) devices and reproducibility of results. This lack of algorithmic openness remains a key limitation in advancing evidence-based use of wearables in clinical and research settings. Future work should prioritize collaboration with manufacturers, adoption of open-source algorithms, and population-specific calibration to improve the accuracy, reproducibility, and trustworthiness of wearable-derived sleep measures. Additionally, only the default “normal” sensitivity mode was tested, and it remains unclear how alternative settings (e.g., “sensitive”) might impact classification accuracy, highlighting the need for future research to compare performance across modes. Second, this study focused solely on nighttime sleep, excluding daytime sleep episodes or napping, which may differ physiologically. In addition, the use of a single night of in-home PSG may not fully capture night-to-night variability in sleep, particularly in individuals with insomnia. While this approach aligns with most validation studies of CST devices, it limits the reliability of assessing typical sleep patterns and device performance across multiple nights. Third, inter-rater variability in PSG scoring may have introduced random error but reflects typical practice and likely led to conservative estimates of agreement. Fourth, although the device’s limitations in detecting wakefulness may vary by sleep pattern, stratified analyses by insomnia severity or the presence of comorbid sleep disorders were not performed because of limited sample sizes. Fifth, the predominantly White sample, with only one African American participant, may restrict generalizability given known sensor performance differences related to skin tone [59].

## 5. Conclusions

This study provides important evidence on the performance of the Fitbit Sense for sleep monitoring in individuals with KOA and comorbid insomnia. While the device performs well in detecting overall sleep under free-living conditions, limitations remain in identifying wakefulness and accurately classifying sleep stages. These findings indicate that, although not appropriate for diagnostic use, the Fitbit Sense may be a useful complementary tool for tracking sleep-derived parameters, including sleep duration, timing, regularity, and chronotype, as well as analyzing individual variability and longitudinal trends at both personal and population levels. The observed variability in device accuracy across the magnitude of nighttime sleep outcomes (e.g., duration of nocturnal awakenings) underscores the importance of personalized application and cautious interpretation.

## Figures and Tables

**Table 1:** Demographics and baseline characteristics of the participants in this study. Categorical variables are reported as frequencies.

Demographics			Mean (SD)/N	Median (IQR)	Range/Percentage
	Age, years		61.0 (8.2)	63 (13.5)	45–78
	Female		32		60.4
	BMI, kg/m^2^		24.7 (2.6)		20.2–30.5
	White-Caucasian		52		98.1
	Postsecondary education		46		86.8
	Currently working		23		43.4
	Lives alone		17		30.1
	Smoking		5		9.3
Baseline characteristics					
	Pain duration, years		9.7 (9.7)	5 (12.7)	0.8–45
	Pain NRS		5.2 (1.9)	5 (3)	2–9
	Pain at night		7.0		86.5
	KOOS pain		54.2 (14.2)		13.9–91.7
	KOOS Function		62.6 (13.9)	63.2 (13.2)	23.5–94.1
	KOOS Quality of life		39.3 (15.7)		0–81.3
	≥1 Comorbidities		45		84.9
	BPI Severity		5 (2.1)	5 (2.3)	1.3–14
	BPI Interference		3.8 (1.8)	3.6 (3.3)	1–7.4
	CSI		38 (12.7)		12–68
	HADS Anxiety		6.3 (3.3)	6 (4)	2–15
		Moderate to severe symptoms	6		12
	HADS Depression		5.2 (3.7)	4.5 (6)	0–16
		Moderate to severe symptoms	4		8
Sleep characteristics					
	Sleep problem duration, years		11.1 (11.0)	8 (14.5)	0.6–54
	Sleep medication use		12		22.6
	ISI		15.8 (4.1)	17 (4)	5–24
		No Insomnia	2		3.9
		Subclinical insomnia	15		29.4
		Clinical insomnia, moderate	31		60.8
		Clinical insomnia, severe	3		5.9
	PSQI		10.2 (2.9)	10 (2)	4–17
		Poor sleep quality (PSQI > 5)	49		96.1
	BFS Mental Fatigue		29.6 (23.6)	25 (41.7)	0–83.3
	BFS Physical Fatigue		34 (21.9)	33.3 (33.3)	0–83.3
	ESS		8.4 (4.1)	8 (4)	1–20
		Excessive daytime sleepiness (ESS > 10)	17		32
	AHI (h^−1^)		9 (9.7)	5.1 (9)	0.5–45.7
		Moderate (AHI ≥ 15)	10		16.1
		Severe (AHI ≥ 30)	3		4.8
	PLMSI (h^−1^)		8.5 (18)	1.3 (7)	0–100.2
		Moderate (PLMSI ≥ 25)	5		8.1
		Severe (PLMSI ≥ 50)	2		1.2

NRS = Numeric Rating Scale; KOOS = Knee Disability and Osteoarthritis Outcome Score; BPI = Brief Pain Inventory; CSI = Central Sensitization Inventory; HADS = Hospital Anxiety and Depression Scale; ISI = Insomnia Severity Index; PSQI = Pittsburgh Sleep Quality Index; BFS = Brugmann Fatigue Scale; ESS = Epworth Sleepiness Scale; AHI = Apnea–Hypopnea Index; PLMSI = Periodic Limb Movement Sleep Index.

**Table 2:** PSG-derived sleep outcomes for the participants in this study.

	Mean (SD)	Median (IQR)	Range
TIB (min)	477 (60)	482 (81)	318–593
TST (min)	373 (63)	387 (76)	213–489
SE (%)	78 (8)	79 (11)	51–91
SOL (min)	20 (18)	17 (20)	1–114
WASO (min)	84 (37)	80 (52)	23–169
Light			
Duration (min)	241 (50)	241 (61)	126–339
Percentage	65 (10)	66 (13)	36–85
Deep			
Duration (min)	47 (30)	45 (38)	2–160
Percentage	13 (8)	11 (10)	1–41
REM			
Duration (min)	85 (30)	86 (38)	0–146
Percentage	22 (6)	23 (7)	0–34

TIB = Total time in bed; TST = Total sleep time; SE = Sleep efficiency; SOL = Sleep onset latency; WASO = Wake after sleep onset; SD = Standard deviation; IQR = Interquartile range.

**Table 3:** Group-level differences between Fitbit Sense and PSG. The positive mean bias indicates overestimation by Fitbit Sense, while the negative mean bias indicates underestimation. The mean absolute difference captures the average deviation magnitude regardless of direction. The standard error represents the variability of the mean bias estimate, with smaller values indicating more consistent measurements across observations.

	Mean Bias ± SD (95% CI)	Mean Absolute Difference	Mean Standard Error (95% CI)
TST (min)	37.05 ± 32.04 (28.97, 45.13)	40.81	0.52 (39.86–41.85)
SE (%)	7.89 ± 6.95 (6.13, 9.65)	8.65	0.11 (8.42–8.88)
SOL (min)	−5.8 ± 22.5 (−11.52, −0.08)	13.1	0.4 (12.3–13.8)
WASO (min)	−31.23 ± 31.45 (−39.22, −23.24)	35.79	0.37 (35.05–36.53)
Light (min)	22.29 ± 53.17 (8.74, 35.84)	45.63	0.67 (44.28–46.98)
Deep (min)	13.74 ± 33.23 (5.34, 22.14)	40.81	0.44 (39.93–41.69)
REM (min)	1.02 ± 36.13 (−10.15, 12.19)	28.08	0.43 (27.21–28.95)

PSG = Polysomnography; TIB = Total time in bed; TST = Total sleep time; SE = Sleep efficiency; WASO = Wake after sleep onset; REM = Rapid-eye-movement; SD = Standard deviation; CI = Confidence interval.

**Table 4:** Group-level discrepancies, bias, and LOAs between Fitbit Sense and PSG. If proportional bias was observed, a linear model was used to predict discrepancies based on the corresponding PSG outcomes, with 95% confidence intervals reported for the model’s intercept (b0) and slope (b1). In cases of heteroscedasticity, another linear model was applied to predict the absolute residuals of the initial model using PSG-derived measures, with 95% confidence intervals provided for the intercept (c0) and slope (c1).

	Fitbit Sense Mean, ±SD	PSG Mean, ±SD	Proportional Bias, 95% CI	LOA Lower, 95% CI	LOA Upper, 95% CI
TST (min)	409.90 ± 56.40	372.85 ± 63.34	123.37 − 0.23 × ref b0 = [79.46, 167.28], b1 = [−0.35, −0.12]	bias − 55.83 bias − [49.46, 64.79]	bias + 55.83 bias + [49.46, 64.79]
SE (%)	85.87 ± 5.41	77.99 ± 8.35	57.54 − 0.64 × ref b0 = [46.69, 68.4], b1 = [−0.78, −0.5]	bias − 8.78 bias − [6.96, 10.98]	bias + 8.78 bias + [6.96, 10.98]
SOL (min)	14.52 ± 17.53	20.34 ± 17.77	10.75 − 0.82 × ref b0 = [4.02, 17.49], b1 = [−1.07, −0.56]	bias − ref × 1.85 bias − ref × [1.7, 1.93]	bias + ref × 1.85 bias + ref × [1.7, 1.93]
WASO (min)	53.00 ± 22.67	84.23 ± 37.50	25.12 − 0.67 × ref b0 = [13.09, 37.16], b1 = [−0.8, −0.54]	bias − 2.46 (7.64 + 0.09 × ref) c0 = [0.91, 14.36], c1 = [0.02, 0.16]	bias + 2.46 (7.64 + 0.09 × ref) c0 = [0.91, 14.36], c1 = [0.02, 0.16]
Light (min)	263.27 ± 48.35	240.98 ± 49.70	166.66 − 0.6 × ref b0 = [110.36, 222.96], b1 = [−0.83, −0.37]	bias − 86.34 bias − [71.84, 103.48]	bias + 86.34 bias + [71.84, 103.48]
Deep (min)	60.90 ± 26.93	47.16 ± 30.10	47.19 − 0.71 × ref b0 = [34.99, 59.38], b1 = [−0.93, −0.49]	bias − 49.92 bias − [41.52, 59.66]	bias + 49.92 bias + [41.52, 59.66]
REM (min)	85.72 ± 35.64	84.70 ± 30.01	45.03 − 0.52 × ref b0 = [19.85, 70.21], b1 = [−0.8, −0.24]	bias − 63.88 bias − [55.04, 75.85]	bias + 63.88 bias + [55.04, 75.85]

TIB = Total time in bed; TST = Total sleep time; SE = Sleep efficiency; SOL = Sleep onset latency, WASO = Wake after sleep onset; REM = Rapid-eye-movement; SD = Standard deviation; PSG = Polysomnography; LOA = Limit of agreement; CI = Confidence interval.

**Table 5:** Group-level proportional error matrix reporting the group average proportion of epochs in each sleep stage as mean (standard deviation) [95% confidence intervals]. Each cell of the matrix displays the average proportion of epochs assigned to each classification category, along with the corresponding standard deviation and 95% confidence intervals. This matrix quantifies the proportion of epochs assigned to each sleep stage by Fitbit Sense relative to PSG.

PSG Stage	Fitbit Sense Wake	Fitbit Sense Light	Fitbit Sense Deep	Fitbit Sense REM
Wake	0.51 (0.15) [0.47, 0.55]	0.37 (0.15) [0.33, 0.4]	0.02 (0.04) [0.01, 0.03]	0.11 (0.1) [0.08, 0.13]
Light	0.04 (0.03) [0.04, 0.05]	0.73 (0.10) [0.7, 0.75]	0.13 (0.07) [0.11, 0.15]	0.10 (0.07) [0.08, 0.12]
Deep	0.02 (0.06) [0.01, 0.03]	0.46 (0.25) [0.39, 0.52]	0.49 (0.27) [0.42, 0.55]	0.03 (0.08) [0.01, 0.05]
REM	0.04 (0.05) [0.02, 0.05]	0.33 (0.25) [0.26, 0.39]	0.03 (0.06) [0.02, 0.05]	0.60 (0.27) [0.54, 0.67]

PSG = Polysomnography; REM = Rapid-eye-movement.

**Table 6:** Group-level EBE metrics assessing Fitbit Sense agreement for each sleep stage detection. EBE agreement metrics are reported for each sleep stage, compared against all other possible classifications, and averaged out for all participants. The Fitbit Sense was evaluated against the corresponding reference epochs from the PSG. Sensitivity represents the percentage of epochs identified as a specific sleep stage by PSG that are correctly detected as that stage by Fitbit Sense. Data is reported as mean (standard deviation) [95% confidence intervals]. Values approaching 1.0 represent higher accuracy for the given metric.

Stage	Accuracy	Sensitivity	Specificity	PPV	NPV	Kappa	PABAK
Wake	85.76 (5.49) [84.44, 87.17]	50.96 (15.46) [47.14, 54.72]	95.95 (2.90) [95.27, 96.71]	76.91 (14.22) [73.49, 80.51]	87.05 (7.08) [85.3, 88.82]	0.51 (0.13) [0.48, 0.55]	0.72 (0.11) [0.69, 0.74]
Light	67.22 (7.85) [65.33, 69.19]	72.50 (9.98) [70.05, 75.07]	62.62 (13.32) [59.36, 65.93]	66.51 (11.98) [63.62, 69.50]	68.87 (10.70) [66.18, 71.48]	0.34 (0.15) [0.31, 0.38]	0.34 (0.16) [0.31, 0.38]
Deep	87.65 (4.57) [86.55, 88.82]	48.75 (27.24) [42.04, 55.61]	91.76 (4.41) [90.70, 92.86]	39.65 (25.94) [33.23, 45.94]	94.44 (4.91) [93.32, 95.73]	0.34 (0.23) [0.28, 0.39]	0.75 (0.09) [0.73, 0.78]
REM	85.23 (6.02) [83.75, 86.74]	60.41 (26.51) [53.82, 67.12]	90.79 (5.27) [89.54, 92.15]	56.62 (18.09) [52.29, 61.15]	91.39 (6) [89.94, 92.91]	0.47 (0.23) [0.41, 0.53]	0.70 (0.12) [0.67, 0.74]

EBE = Epoch-by-epoch; PSG = Polysomnography; REM = Rapid-eye-movement; PPV = Positive predictive value; NPV = Negative predictive value; PABAK = Prevalence and bias-adjusted kappa.

## Data Availability

All data are available upon reasonable request to the corresponding author.

## Supplementary Materials

The following supporting information can be downloaded at: https://www.mdpi.com/article/10.3390/s25154813/s1, Supplementary Table S1: Number of participants for each outcome measure; Supplementary Table S2: Group-level absolute error matrix; Supplementary Figure S1: Individual EBE metrics for each considered stage represented in boxplots; Supplementary Table S3: Group-level absolute error matrix assessing Fitbit Sense agreement for each sleep stage detection; Supplementary Table S4: Group-level discrepancies, bias and LOAs between Fitbit Sense and PSG for 64 nights; Supplementary Figure S2: Bland–Altman plots of the sleep outcomes for Fitbit Sense–PSG comparison for each sleep stage; Supplementary Table S5: Group-level proportional error matrix reporting the group average proportion of epochs in each sleep stage; Supplementary Table S6: Group-level EBE metrics assessing Fitbit Sense agreement for each sleep stage detection; Supplementary Table S7: Group-level discrepancies, bias and LOAs between Fitbit Sense and PSG for 64 nights; Supplementary Figure S3: Bland–Altman plots of the sleep outcomes for Fitbit Sense–PSG comparison for each sleep stage; Supplementary Table S8: Group-level proportional error matrix reporting the group average proportion of epochs in each sleep stage; Supplementary Table S9: Group-level EBE metrics assessing Fitbit Sense agreement for each sleep stage detection; Supplementary Table S10: baseline characteristics comparison between included and excluded participants.

## Abbreviations

The following abbreviations are used in this manuscript:

OA	Osteoarthritis
PSG	Polysomnography
CST	Consumer sleep tracking
RCT	Randomized controlled trial
TIB	Time in bed
EBE	Epoch-by-epoch
EEG	Electroencephalography
EOG	Electrooculography
EMG	Electromyography
TST	Total sleep time
SE	Sleep efficiency
SOL	Sleep onset latency
WASO	Wake after sleep onset
SD	Standard deviation
IQR	Interquartile range
LOAs	Limits of agreement
PABAK	Prevalence-adjusted bias-adjusted kappa
NRS	Numeric Rating Scale
KOOS	Knee Disability and Osteoarthritis Outcome Score
BPI	Brief Pain Inventory
CSI	Central Sensitization Inventory
HADS	Hospital Anxiety and Depression Scale
PSQI	Pittsburgh Sleep Quality Index
ISI	Insomnia Severity Index
BFS	Brugmann Fatigue Scale
ESS	Epworth Sleepiness Scale
AHI	Apnea–Hypopnea Index
PLMSI	Periodic Limb Movement Sleep Index
REM	Rapid-eye-movement
CI	Confidence interval
