# Supplementary material for: Can a Commercially Available Smartwatch Device Accurately Measure Nighttime Sleep Outcomes in Individuals with Knee Osteoarthritis and Comorbid Insomnia? A Comparison with Home-Based Polysomnography
Source: Sensors (Basel). 2025 Aug 5;25(15):4813. doi: 10.3390/s25154813 (PMC12349529; doi:10.3390/s25154813)
Supplement: Supplementary file 1 [file sensors-25-04813-s001.zip › sensors-3729126-supplementary.pdf]

Supplementary Material

Can a Commercially Available Smartwatch Device Accurately Measure Nighttime Sleep Outcomes in Individuals with Knee Osteoarthritis and Comorbid Insomnia? A Comparison with Home-Based Polysomnography

Céline Labie [1,2], Nils Runge [1,2,3], Zosia Goossens [2,3], Olivier Mairesse [3], Jo Nijs [2,4,5], Anneleen Malfliet [2,6,7], Dieter Van Assche [1,8], Kurt de Vlam [8,9], Luca Menghini [10,11], Sabine Verschueren [1,†] and Liesbet De Baets [1,2,12,13,*,†]

[1] Musculoskeletal Rehabilitation Research Group, Department of Rehabilitation Sciences, Faculty of Movement and Rehabilitation Sciences, KU Leuven, 3001 Leuven, Belgium
[2] Pain in Motion Research Group (PAIN), Department of Physiotherapy, Human Physiology and Anatomy, Faculty of Physical Education and Physiotherapy, Vrije Universiteit Brussel, 1050 Brussels, Belgium
[3] Brain, Body and Cognition, Faculty of Psychology and Educational Sciences, Vrije Universiteit Brussel, 1090 Brussels, Belgium
[4] PijnPraxis.be Private Practice for Pain Physiotherapy, 3970 Leopoldsburg, Belgium
[5] Unit of Physiotherapy, Department of Health and Rehabilitation, Institute of Neuroscience and Physiology, Sahlgrenska Academy, University of Gothenburg, SE-405 30 Gothenburg, Sweden
[6] Chronic Pain Rehabilitation, Department of Physical Medicine and Physiotherapy, University Hospital Brussels, 1050 Brussels, Belgium
[7] Research Foundation Flanders, 1000 Brussels, Belgium
[8] Division of Rheumatology, University Hospitals Leuven, 3000 Leuven, Belgium
[9] Skeletal Biology & Engineering Research Center, Department of Development and Regeneration, KU Leuven, 3000 Leuven, Belgium
[10] Department of General Psychology, University of Padova, 35131 Padova, Italy
[11] Human Inspired Technology Research Centre, University of Padova, 35131 Padova, Italy
[12] Department of Physical and Rehabilitation Medicine, University Hospitals Leuven, 3212 Leuven, Belgium
[13] Leuven Algologic Center, University Hospitals Leuven, 3212 Leuven, Belgium
* Correspondence: liesbet.debaets@kuleuven.be
† These authors contributed equally to this work.

Contents

Supplementary Material Table S1 ... 4

Number of participants for each outcome measure ... 4

Supplementary Material Table S2 ... 5

Group-level absolute error matrix ... 5

Supplementary Material Figure S1 ... 6

Detailed visualization of individual-level EBE metrics ... 6

Supplementary Material Table S3 ... 7

Group-level EBE metrics based on the absolute error matrix ... 7

Supplementary Material Table S4 ... 8

1.1 Discrepancy analysis ... 8

1.1.1 Group-level discrepancies, bias, and LOAs ... 8

Supplementary Material Figure S2 ... 9

1.1 Discrepancy analysis ... 9

1.1.2 Bland–Altman plots of the sleep outcomes for Fitbit Sense–PSG ... 9

Supplementary Material Table S5 ... 10

1.2 Epoch-by-epoch analysis ... 10

1.2.1 EBE proportional error matrix ... 10

Supplementary Material Table S6 ... 11

First sensitivity analysis of 64 nights, including all data points with outliers ... 11

1.2 Epoch-by-epoch analysis ... 11

1.2.2 Group-level EBE metrics ... 11

Supplementary Table S7 ... 12

2.1 Discrepancy analysis ... 12

2.1.1 Group-level discrepancies, bias, and LOAs ... 12

Supplementary Figure S3 ... 13

2.1 Discrepancy analysis ... 13

2.1.2 Bland–Altman plots of the sleep outcomes for Fitbit Sense–PSG ... 13

Supplementary Table S8 ... 15

2.2 Epoch-by-epoch analysis ... 15

2.2.1 EBE proportional error matrix ... 15

Supplementary Table S9 ... 15

2.2 Epoch-by-epoch analysis ... 15

2.2.2 Group-level EBE metrics ... 15

Third sensitivity analysis comparing baseline characteristics between included and excluded participants 16

Supplementary Material Table S1

Number of participants for each outcome measure

Outcome	Number of participants
Demographics	53
**Baseline characteristics**	
Pain duration, years	53
Pain NRS	49
Pain at night	52
KOOS pain	51
KOOS Function	51
KOOS Quality of life	51
≥ 1 Comorbidities	53
BPI Severity	52
BPI Interference	52
CSI	50
HADS Anxiety	52
HADS Depression	52
**Sleep characteristics**	
Sleep problem duration, years	53
Sleep medication use	53
ISI	51
PSQI	51
BFS Mental Fatigue	51
BFS Physical Fatigue	51
ESS	50
	**Number of nights**
AHI (h^{-1})	62
PLMSI (h^{-1})	62

Supplementary Table S1 Number of participants for each outcome measure.
NRS = Numeric Rating Scale; KOOS = Knee Disability and Osteoarthritis Outcome Score; BPI = Brief Pain Inventory; CSI = Central Sensitization Inventory; HADS = Hospital Anxiety and Depression Scale; ISI = Insomnia Severity Index; PSQI = Pittsburgh Sleep Quality Index; BFS = Brugmann Fatigue Scale; ESS = Epworth Sleepiness Scale; AHI = Apnea–Hypopnea Index; PLMSI = Periodic Limb Movement Sleep Index

Supplementary Material Table S2

Group-level absolute error matrix

PSG Stage	Fitbit Sense wake	Fitbit Sense light	Fitbit Sense deep	Fitbit Sense REM	PSG Total
wake	6486	4964	209	1307	12966
light	1373	21599	3962	2948	29882
deep	101	2543	3035	169	5848
REM	412	3540	346	6205	10503
Fitbit Sense Total	8372	32646	7552	10629	59199

Supplementary Table S2 Group-level absolute error matrix. Each cell of the matrix displays the number of epochs in each condition.
PSG = Polysomnography; REM = Rapid-eye-movement

Supplementary Material Figure S1

Detailed visualization of individual-level EBE metrics

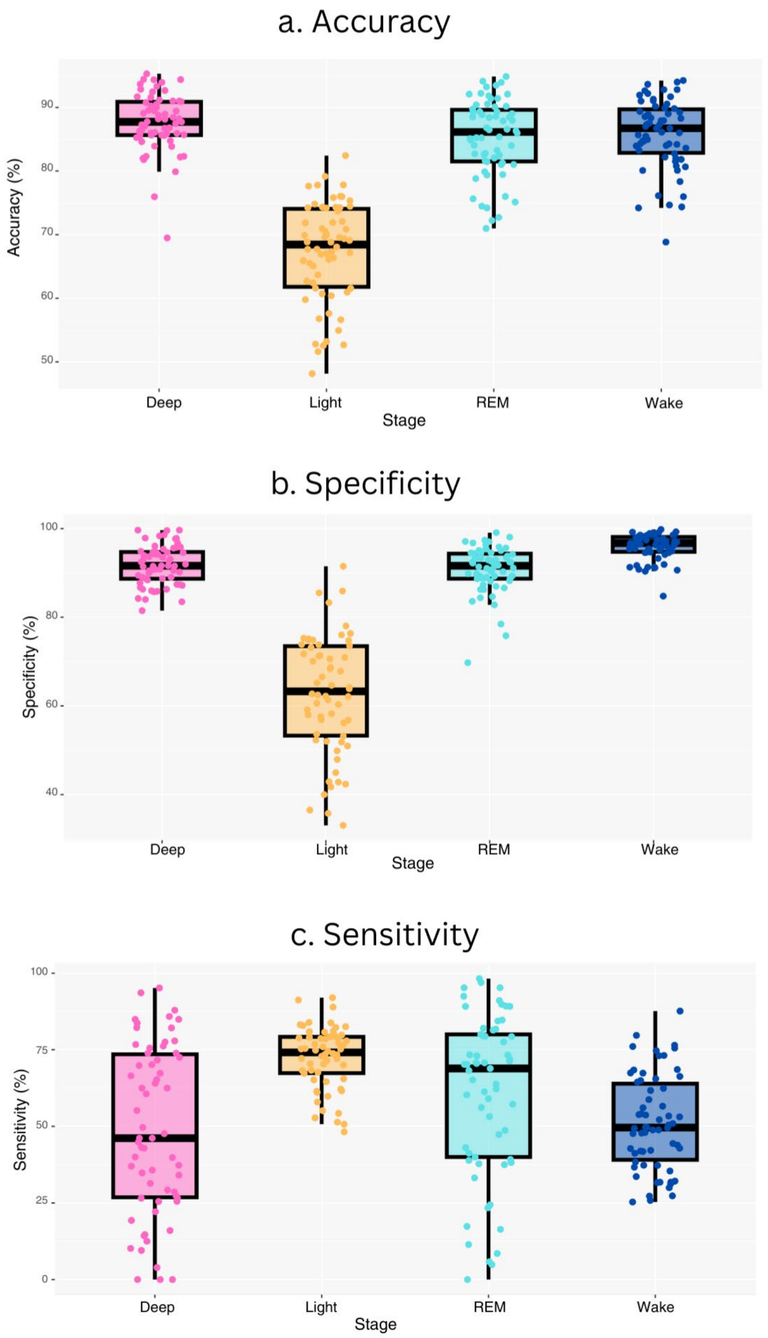

Supplementary Figure S1 Individual EBE metrics for each considered stage represented in boxplots. Sensitivity represents the percentage of epochs identified as a specific sleep stage by PSG that are correctly detected as that stage by Fitbit Sense.
EBE= Epoch-by-epoch; PSG = Polysomnography; REM = Rapid-eye-movement

Supplementary Material Table S3

Group-level EBE metrics based on the absolute error matrix

Stage	Accuracy	Sensitivity	Specificity	PPV	NPV	Kappa	PABAK
Wake	85.87	50.02	95.92	77.47	87.25	0.53	0.72
Light	67.35	72.28	62.32	66.16	68.80	0.35	0.35
Deep	87.62	51.90	91.53	40.18	94.55	0.38	0.75
REM	85.27	59.08	90.92	58.39	91.15	0.50	0.71

Supplementary Table S3 Group-level absolute error matrix assessing Fitbit Sense agreement for each sleep stage detection. EBE agreement metrics are reported for each sleep stage, based on the absolute error matrix using the total sum of epochs in each condition, without distinguishing between subjects.

EBE= Epoch-by-epoch; REM = Rapid-eye-movement; PPV = Positive predictive value; NPV = Negative predictive value; PABAK = Prevalence and bias-adjusted kappa

Supplementary Material Table S4

First sensitivity analysis of 64 nights, including all data points with outliers

1.1 Discrepancy analysis

1.1.1 Group-level discrepancies, bias, and LOAs

	Fitbit Sense Mean, ± SD	PSG Mean, ± SD	Proportional Bias, 95% CI	LOA Lower, 95% CI	LOA Upper, 95% CI
TST (min)	404.90 ± 62.27	371.84 ± 62.96	107.86 - 0.2 x ref<br>b0 = [52.04, 163.69], b1 = [-0.35, -0.05]	bias - ref x 0.23<br>bias - ref x [0.18, 0.28]	bias + ref x 0.23<br>bias + ref x [0.18, 0.28]
SE (%)	85.13 ± 6.77	78.19 ± 8.37	62.59 - 0.71 x ref<br>b0 = [47.5, 77.68], b1 = [-0.9, -0.52]	bias - ref x 0.23<br>bias - ref x [0.18, 0.28]	bias + ref x 0.23<br>bias + ref x [0.18, 0.28]
SOL (min)	16.34 ± 21.23	20.23 ± 17.57	13.76 - 0.87 x ref<br>b0 = [5.61, 21.90], b1 = [-1.18, -0.57]	bias - ref x 1.86<br>bias - ref x [1.72, 1.93]	bias + ref x 1.86<br>bias + ref x [1.72, 1.93]
WASO (min)	53.82 ± 23.96	82.98 ± 37.63	13.76 - 0.87 x ref<br>b0 = [15.4, 41.16], b1 = [-0.83, -0.55]	bias - 41.12<br>bias - [32.62, 51.46]	bias + 41.12<br>bias + [32.62, 51.46]
Light (min)	259.82 ± 51.37	240.76 ± 48.92	160.92 - 0.59 x ref<br>b0 = [100.66, 221.18], b1 = [-0.83, -0.34]	bias - 92.67<br>bias - [77.67, 111.30]	bias + 92.67<br>bias + [77.67, 111.30]
Deep (min)	60.13 ± 26.85	48.3 ± 31.15	48.91 - 0.77 x ref<br>b0 = [36.83, 61], b1 = [-0.98, -0.56]	bias - 50.68<br>bias - [42.51, 60.36]	bias + 50.68<br>bias + [42.51, 60.36]
REM (min)	84.95 ± 35.47	82.79 ± 31.42	45.89 - 0.53 x ref<br>b0 = [22.85, 68.93], b1 = [-0.79, -0.27]	bias - 63.16<br>bias - [54.55, 74.49]	bias + 63.16<br>bias + [54.55, 74.49]

Supplementary Table S4. Group-level discrepancies, bias, and LOAs between Fitbit Sense and PSG for 64 nights. If proportional bias was observed, a linear model was used to predict discrepancies based on the corresponding PSG outcomes, with 95% confidence intervals reported for the model's intercept (b0) and slope (b1). In cases of heteroscedasticity, another linear model was applied to predict the absolute residuals of the initial model using PSG-derived measures, with 95% confidence intervals provided for the intercept (c0) and slope (c1).

TIB = Total time in bed; TST = Total sleep time; SE = Sleep efficiency; SOL = Sleep onset latency; WASO = Wake after sleep onset; REM = Rapid-eye-movement; SD = Standard deviation; PSG = Polysomnography; LOA = Limit of agreement; CI = Confidence interval

Supplementary Material Figure S2

First sensitivity analysis of 64 nights, including all data points with outliers

1.1 Discrepancy analysis

1.1.2 Bland–Altman plots of the sleep outcomes for Fitbit Sense–PSG

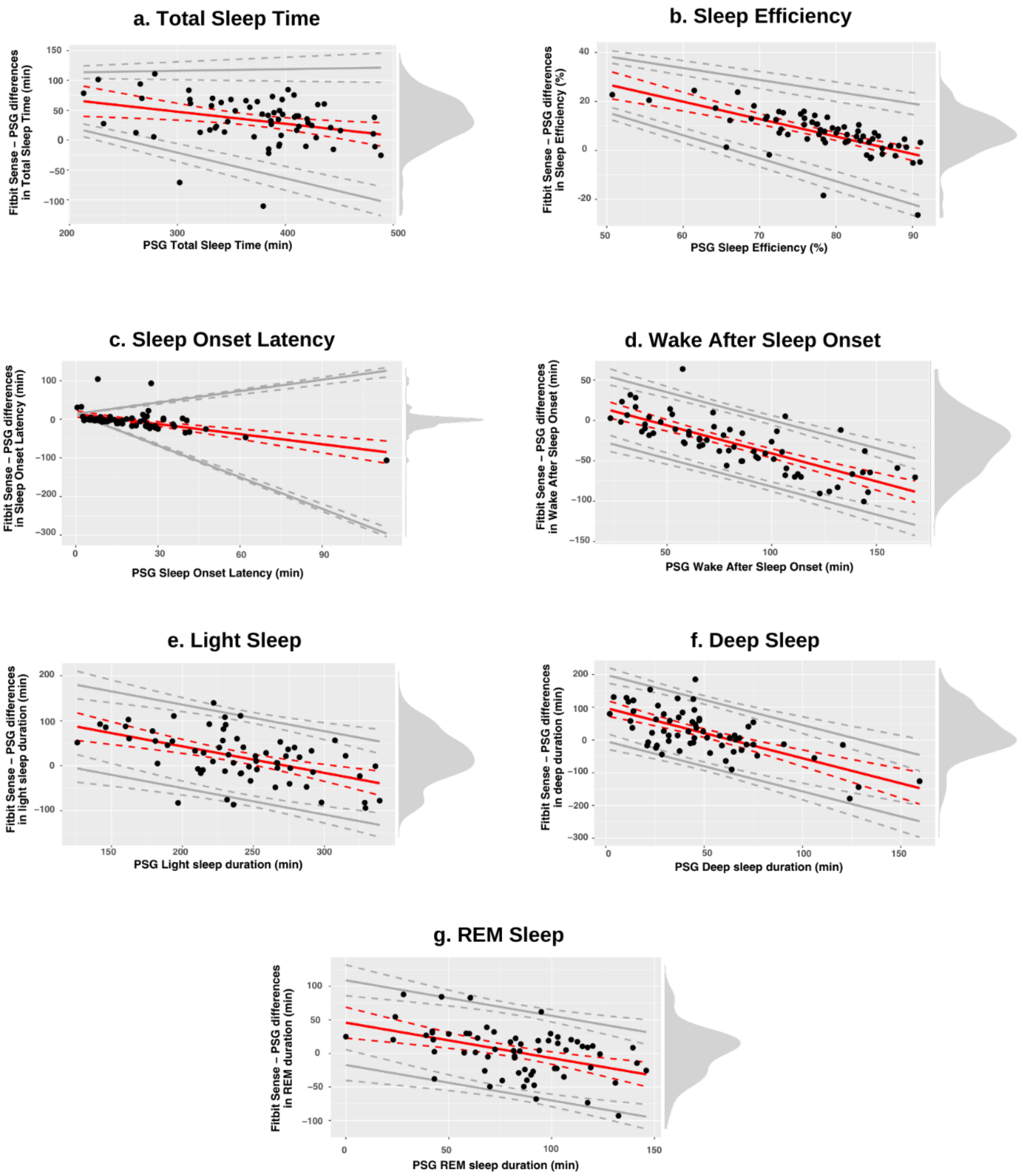

Supplementary Figure S2 Bland–Altman plots of the sleep outcomes for Fitbit Sense–PSG comparison for each sleep stage. Red solid lines indicate bias, whereas gray solid lines indicate the 95% LOAs, both with their 95% CIs (dotted lines). Black points indicate individual observations, and the density diagram on the right side of each plot represents the distribution of

the Fitbit Sense–PSG differences. The zero point on the y-axis denotes perfect agreement between the two methods, while values above and below zero indicate overestimation and underestimation, respectively, in comparison to PSG. A diagonal trend in the mean bias line indicates a considerable proportional bias, whereas diverging limits of agreement suggest notable heteroscedasticity.
PSG = Polysomnography; REM = Rapid-eye-movement

Supplementary Material Table S5

First sensitivity analysis of 64 nights, including all data points with outliers

1.2 Epoch-by-epoch analysis

1.2.1 EBE proportional error matrix

**PSG Stage**	**Fitbit Sense Wake**	**Fitbit Sense Light**	**Fitbit Sense Deep**	**Fitbit Sense REM**
Wake	0.52 (0.17) [0.48, 0.56]	0.36 (0.16) [0.32, 0.39]	0.02 (0.04) [0.01, 0.03]	0.11 (0.1) [0.08, 0.13]
Light	0.05 (0.05) [0.04, 0.06]	0.72 (0.11) [0.69, 0.74]	0.13 (0.07) [0.11, 0.14]	0.10 (0.07) [0.09, 0.12]
Deep	0.03 (0.08) [0.01, 0.04]	0.45 (0.25) [0.39, 0.51]	0.49 (0.27) [0.42, 0.55]	0.03 (0.08) [0.01, 0.05]
REM	0.05 (0.13) [0.01, 0.08]	0.32 (0.25) [0.25, 0.38]	0.03 (0.06) [0.02, 0.05]	0.6 (0.28) [0.53, 0.67]

Supplementary Table S5 Group-level proportional error matrix reporting the group average proportion of epochs in each sleep stage as mean (standard deviation) [95% confidence intervals]. Each cell of the matrix displays the average proportion of epochs assigned to each classification category, along with the corresponding standard deviation and 95% confidence intervals. This matrix quantifies the proportion of epochs assigned to each sleep stage by Fitbit Sense relative to PSG.
PSG = Polysomnography; REM = Rapid-eye-movement

Supplementary Material Table S6

First sensitivity analysis of 64 nights, including all data points with outliers

1.2 Epoch-by-epoch analysis

1.2.2 Group-level EBE metrics

Stage	Accuracy	Sensitivity	Specificity	PPV	NPV	Kappa	PABAK
Wake	85.41 (5.78) [84.03, 86.9]	52.15 (16.63) [48.16, 56.19]	95.20 (5.10) [94.09, 96.56]	75.64 (15.91) [71.75, 79.70]	87.38 (7.21) [85.69, 89.19]	0.51 (0.14) [0.48, 0.54]	0.71 (0.12) [0.68, 0.74]
Light	67.13 (7.81) [65.24, 69.03]	71.85 (10.52) [69.29, 74.37]	63.23 (13.62) [59.80, 66.55]	66.92 (12.16) [64.05, 69.87]	68.43 (10.82) [65.84, 71.09]	0.34 (0.15) [0.31, 0.38]	0.34 (0.16) [0.31, 0.38]
Deep	87.49 (5.03) [86.32, 88.77]	48.55 (27.27) [41.94, 55.26]	91.90 (4.41) [90.83, 92.99]	40.48 (26.04) [34.10, 46.80]	94.13 (5.56) [92.85, 95.57]	0.34 (0.23) [0.28, 0.39]	0.75 (0.10) [0.73, 0.78]
REM	85.22 (5.94) [83.82, 86.67]	60.08 (27.64) [53.50, 67.05]	90.67 (5.23) [89.46, 91.98]	55.31 (19.43) [50.67, 60.33]	91.55 (6.00) [90.1, 93.05]	0.46 (0.24) [0.40, 0.52]	0.70 (0.12) [0.68, 0.73]

Supplementary Table S6 Group-level EBE metrics assessing Fitbit Sense agreement for each sleep stage detection. EBE agreement metrics are reported for each sleep stage, compared against all other possible classifications, and averaged out for all participants. The Fitbit Sense was evaluated against the corresponding reference epochs from the PSG. Sensitivity represents the percentage of epochs identified as a specific sleep stage by PSG that are correctly detected as that stage by Fitbit Sense. Data are reported as mean (standard deviation) [95% confidence intervals]. Values approaching 1.0 represent higher accuracy for the given metric.

EBE= Epoch-by-epoch; PSG = Polysomnography; REM = Rapid-eye-movement; PPV = Positive predictive value; NPV = Negative predictive value; PABAK = Prevalence and bias-adjusted kappa

Supplementary Table S7

Second sensitivity analysis of 54 nights, including participants with data from a single night

2.1 Discrepancy analysis

2.1.1 Group-level discrepancies, bias, and LOAs

	Fitbit Sense Mean, ± SD	PSG Mean, ± SD	Proportional Bias, 95% CI	LOA Lower, 95% CI	LOA Upper, 95% CI
TST (min)	414.11 ± 54.82	376.43 ± 63.15	134.93 - 0.26 x ref<br>b0 = [87.01, 182.86], b1 = [-0.38, -0.13]	bias - 55.86<br>bias - [49.22, 65.66]	bias + 55.86<br>bias + [49.22, 65.66]
SE (%)	85.76 ± 5.58	77.83 ± 8.66	56.70 - 0.63 x ref<br>b0 = [45.26, 68.14], b1 = [-0.77, -0.48]	bias - 8.91<br>bias - [6.88, 11.40]	bias + 8.91<br>bias + [6.88, 11.40]
SOL (min)	15.05 ± 18.17	20.84 ± 18.47	12.05 - 0.86 x ref<br>b0 = [4.54, 19.56], b1 = [-1.13, -0.59]	bias - 35.23<br>bias - [17.33, 54.14]	bias + 35.23<br>bias + [17.33, 54.14]
WASO (min)	53.94 ± 23.77	85.83 ± 37.63	24.81 - 0.66 x ref<br>b0 = [11.5, 38.12], b1 = [-0.80, -0.52]	bias - 2.46(8.42 + 0.09 x ref)<br>c0 = [0.97, 15.88], c1 = [0.01, 0.17]	bias + 2.46(8.42 + 0.09 x ref)<br>c0 = [0.97, 15.88], c1 = [0.01, 0.17]
Light (min)	267.82 ± 49.62	244.09 ± 51.55	172.01 - 0.61 x ref<br>b0 = [111.03, 232.99], b1 = [-0.85, -0.36]	bias - 88.79<br>bias - [72.63, 107.96]	bias + 88.79<br>bias + [72.63, 107.96]
Deep (min)	61.99 ± 26.17	48.02 ± 30.82	49.55 - 0.74 x ref<br>b0 = [36.74, 62.36], b1 = [-0.97, -0.52]	bias - 48.86<br>bias - [39.41, 59.59]	bias + 48.86<br>bias + [39.41, 59.59]
REM (min)	84.30 ± 34.39	84.31 ± 29.67	53.97 - 0.64 x ref<br>b0 = [26.59, 81.35], b1 = [-0.95, -0.33]	bias - 64.08<br>bias - [55.62, 76.36]	bias + 64.08<br>bias + [55.62, 76.36]

Supplementary Table S7 Group-level discrepancies, bias, and LOAs between Fitbit Sense and PSG for 64 nights. If proportional bias was observed, a linear model was used to predict discrepancies based on the corresponding PSG outcomes, with 95% confidence intervals reported for the model's intercept (b0) and slope (b1). In cases of heteroscedasticity, another linear model was applied to predict the absolute residuals of the initial model using PSG-derived measures, with 95% confidence intervals provided for the intercept (c0) and slope (c1).

TIB = Total time in bed; TST = Total sleep time; SE = Sleep efficiency; SOL = Sleep onset latency; WASO = Wake after sleep onset; REM = Rapid-eye-movement; SD = Standard deviation; PSG = Polysomnography; LOA = Limit of agreement; CI = Confidence interval

Supplementary Figure S3

Second sensitivity analysis of 54 nights, including participants with data from a single night

2.1 Discrepancy analysis

2.1.2 Bland–Altman plots of the sleep outcomes for Fitbit Sense–PSG

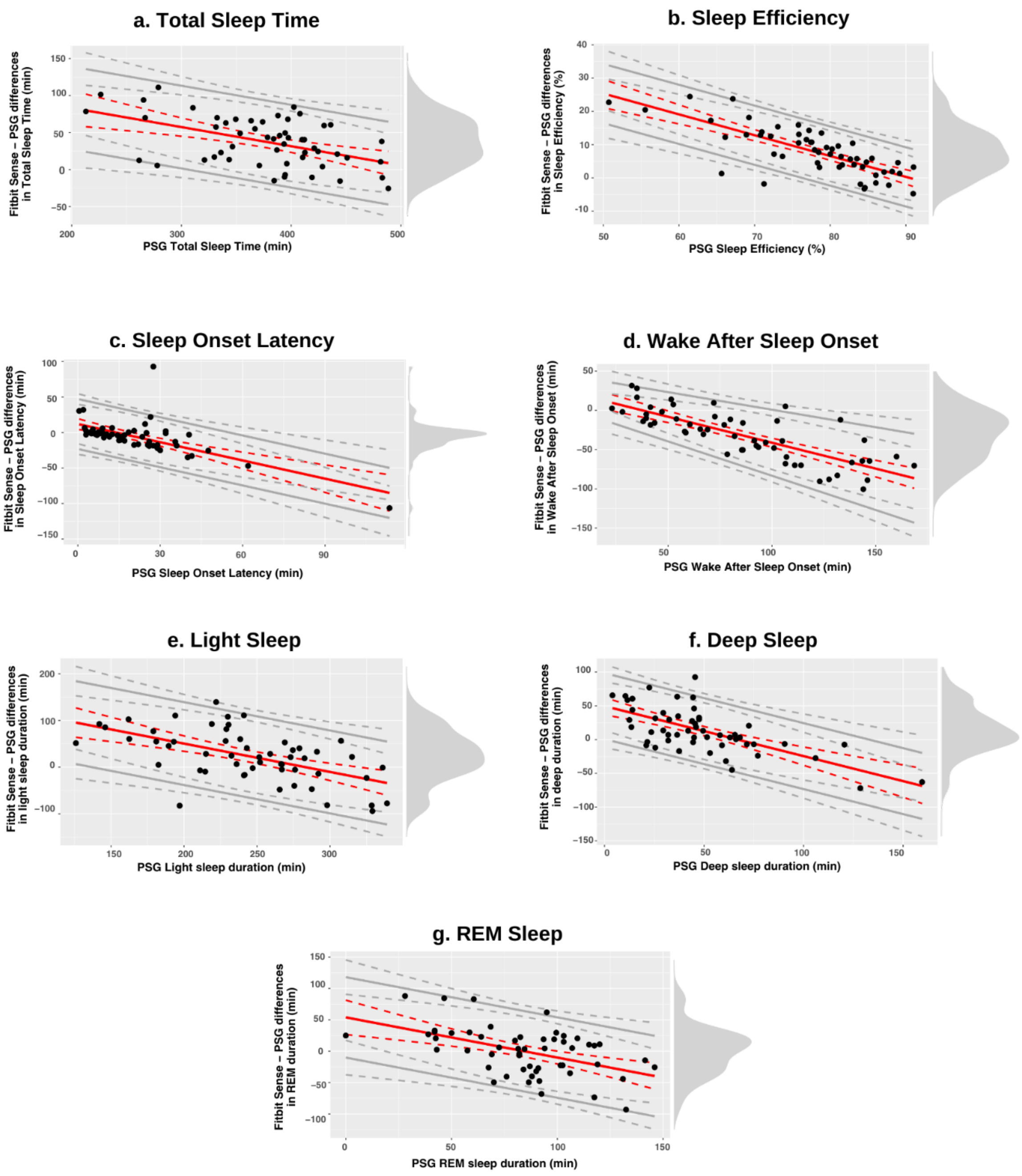

Supplementary Figure S3 Bland–Altman plots of the sleep outcomes for Fitbit Sense–PSG comparison for each sleep stage. Red solid lines indicate bias, whereas gray solid lines indicate the 95% LOAs, both with their 95% CIs (dotted lines). Black points indicate individual observations, and the density diagram on the right side of each plot represents the distribution of

the Fitbit Sense–PSG differences. The zero point on the y-axis denotes perfect agreement between the two methods, while values above and below zero indicate overestimation and underestimation, respectively, in comparison to PSG. A diagonal trend in the mean bias line indicates a considerable proportional bias, whereas diverging limits of agreement suggest notable heteroscedasticity.

PSG = Polysomnography; REM = Rapid-eye-movement

Supplementary Table S8

Second sensitivity analysis of 54 nights, including participants with data from a single night

2.2 Epoch-by-epoch analysis

2.2.1 EBE proportional error matrix

PSG Stage	Fitbit Sense Wake	Fitbit Sense Light	Fitbit Sense Deep	Fitbit Sense REM
Wake	0.51 (0.15) [0.47, 0.55]	0.36 (0.15) [0.32, 0.40]	0.02 (0.04) [0.01, 0.03]	0.10 (0.10) [0.08, 0.13]
Light	0.04 (0.03) [0.04, 0.05]	0.73 (0.10) [0.7, 0.75]	0.13 (0.07) [0.11, 0.15]	0.10 (0.07) [0.08, 0.11]
Deep	0.02 (0.06) [0.01, 0.04]	0.45 (0.24) [0.39, 0.51]	0.49 (0.27) [0.42, 0.56]	0.04 (0.09) [0.01, 0.06]
REM	0.03 (0.04) [0.02, 0.04]	0.33 (0.25) [0.26, 0.39]	0.03 (0.06) [0.02, 0.04]	0.61 (0.27) [0.54, 0.68]

Supplementary Table S8 Group-level proportional error matrix reporting the group average proportion of epochs in each sleep stage as mean (standard deviation) [95% confidence intervals]. Each cell of the matrix displays the average proportion of epochs assigned to each classification category, along with the corresponding standard deviation and 95% confidence intervals. This matrix quantifies the proportion of epochs assigned to each sleep stage by Fitbit Sense relative to PSG.
PSG = Polysomnography; REM = Rapid-eye-movement

Supplementary Table S9

Second sensitivity analysis of 54 nights, including participants with data from a single night

2.2 Epoch-by-epoch analysis*2.2.2 Group-level EBE metrics*

Stage	Accuracy	Sensitivity	Specificity	PPV	NPV	Kappa	PABAK
Wake	85.79 (5.75) [84.32, 87.37]	51.30 (15.38) [47.14, 55.32]	95.99 (3.02) [95.24, 96.81]	77.42 (14.37) [73.73, 81.27]	87.00 (7.34) [85.11, 88.99]	0.52 (0.14) [0.48, 0.55]	0.72 (0.12) [0.69, 0.75]
Light	67.14 (7.97) [65.07, 69.28]	72.68 (9.66) [70.17, 75.30]	62.46 (13.30) [59.04, 65.95]	66.45 (12.24) [63.20, 69.69]	68.74 (11.07) [65.82, 71.70]	0.34 (0.15) [0.30, 0.38]	0.34 (0.16) [0.30, 0.39]
Deep	87.47 (4.62) [86.30, 88.76]	48.72 (26.82) [41.61, 55.72]	91.65 (4.35) [90.50, 92.80]	38.42 (25.95) [31.70, 45.16]	94.35 (5.04) [93.11, 95.76]	0.33 (0.23) [0.27, 0.39]	0.75 (0.09) [0.73, 0.78]
REM	85.46 (6.25) [83.84, 87.12]	60.64 (26.81) [53.58, 67.79]	91.07 (5.30) [89.75, 92.54]	56.80 (18.93) [51.95, 61.92]	91.49 (6.28) [89.88, 93.20]	0.47 (0.23) [0.41, 0.53]	0.71 (0.13) [0.68, 0.74]

Supplementary Table S9 Group-level EBE metrics assessing Fitbit Sense agreement for each sleep stage detection. EBE agreement metrics are reported for each sleep stage, compared against all other possible classifications, and averaged out for all participants. The Fitbit Sense was evaluated against the corresponding reference epochs from the PSG. Sensitivity represents the percentage of epochs identified as a specific sleep stage by PSG that are correctly detected as that stage by Fitbit Sense. Data are reported as mean (standard deviation) [95% confidence intervals]. Values approaching 1.0 represent higher accuracy for the given metric.
EBE = Epoch-by-epoch; PSG = Polysomnography; REM = Rapid-eye-movement; PPV = Positive predictive value; NPV = Negative predictive value; PABAK = Prevalence and bias-adjusted kappa

Supplementary Material Table S10

Third sensitivity analysis comparing baseline characteristics between included and excluded participants

	Excluded	Included	Mean group difference (95% CI)	p-value
**Age**	64.59 (8.04)	60.67 (8.35)	3.91 (0.56 - 7.26)	0.02[a]
**Seks**	M = 15 F = 26	M = 22 F = 33	n. a.	0.90[b]
**BMI**	24.67 (2.55)	24.70 (2.56)	-0.03 (-1.09 - 1.02)	0.95[a]
**KOOS Pain**	57.71 (14.5)	53.51 (14.4)	4.20 (-1.09 - 1.02)	0.18[a]
**PSQI Sleep**	9.69 (3.38)	10.21 (2.92)	-0.52 (-1.81 - 0.85)	0.41[c]

Supplementary Table S10 Baseline characteristics comparison between included and excluded participants. Continuous variables are reported as mean (SD). Categorical variables are reported as frequencies.

[a] Group differences evaluated using the Welch two-sample t-test. [b] Group differences evaluated using Pearson's chi-squared test. [c] Group differences evaluated using the Wilcoxon rank sum test.
BMI = Body Mass Index: KOOS = Knee Injury and Osteoarthritis Outcome Score; PSQI = Pittsburgh Sleep Quality Index; F = Female; M = Male; CI = Confidence Interval; n. a. = Not Applicable.
